# Integrating Genomic Data with Transcriptomic Data for Improved Survival Prediction for Adult Diffuse Glioma

**DOI:** 10.7150/jca.44032

**Published:** 2020-04-06

**Authors:** Qi Yang, Yi Xiong, Nian Jiang, Fanyuan Zeng, Chunhai Huang, Xuejun Li

**Affiliations:** 1 Department of Neurosurgery, Xiangya Hospital, Central South University, No. 87, Xiangya Road, Changsha, Hunan 410008 P. R. China; 2 Hunan International Scientific and Technological Cooperation Base of Brain Tumor Research, Xiangya Hospital, Central South University, No. 87, Xiangya Road, Changsha, Hunan 410008 P. R. China; 3 Department of Neurosurgery, First Affiliated Hospital of Jishou University, Jishou, Hunan, 416000 P. R. China; 4 Centre for Clinical and Translational Medicine Research, Jishou University, Jishou, Hunan, 416000 P. R. China

**Keywords:** glioblastoma, diffused glioma, driver gene mutations, transcriptome, prognosis prediction

## Abstract

**Background**: Glioma is the most common type of primary central nervous system tumors. However, the relationship between gene mutations and transcriptome is unclear in diffuse glioma, and there are no systemic analyses with regard to the genotype-phenotype association currently.

**Methods**: We performed the multi-omics analysis in large glioblastoma multiforme (GBM, n=126) and low-grade glioma (LGG, n=481) cohorts obtained from The Cancer Genome Atlas (TCGA) database. We used multivariate linear models to evaluate associations between driver gene mutations and global gene expression. We developed generalized linear models to evaluate associations between genetic/expression factors with clinicopathologic features. Multivariate Cox proportional hazards models were used to predict the overall survival.

**Results**: The potential relationship between genotype and genetics, clinical as well as pathologic features, on diffused glioma was observed. At least one driver mutation correlated with expression changes of about 10% of genes in GBMs while about 80% of genes in LGGs. The strongest association between mutations and expression changes was observed for* DRG2* and* LRCC41* gene in GBMs and LGGs, respectively. Additionally, the association between genomics features and clinicopathologic features suggested the different underlying molecular mechanisms in molecular subtypes or histology subtypes. For predicting survival, among genetics, transcriptome and clinical variables, transcriptome features made the largest contribution. By combining all the available data, the accuracy in predicting the prognosis of diffuse glioma in patients was also improved.

**Conclusion**: Our study results revealed the influences of driver gene mutations on global gene expression in diffuse glioma patients. A more accurate model in predicting the prognosis of patients was achieved when combining with all the available data than just transcriptomic data.

## Introduction

Glioma is the most common type of primary central nervous system tumors and is divided into two major subgroups based on their infiltrative behavior, circumscribed and diffused 1. The patients with diffuse glioma need total surgical resection and extended resection at times, even though these patients have a worse prognosis when compared to those with circumscribed glioma. Diffuse gliomas have a strong tendency to invade the brain via the white matter. Targeted medicine have improved the prognosis of some patients with diffuse gliomas, but the five-year survival rate still remained very low among all types of it and worsened with the elevation of WHO grades 2, 3.

The current WHO grading system and pathology-based grading do not comprehensively reflect the biological behavior and clinical outcomes in patients with diffuse glioma. Therefore, it is imperative to find more accurate biomarkers that assist in predicting the prognosis of patients. Molecular subgroups of diffuse glioma have been established based on the transcriptomic data and these subgroups were considered to be superior in predicting the prognosis than the WHO grading system 4-6.

The characteristics of glioblastoma multiforme (GBM) and low-grade gliomas (LGG) are distinct clinically, histologically, and genetically. GBM comprises of 46.6% malignant tumors in the central nervous system is highly infiltrative and aggressive when compared to LGG 7-9. Unlike many other types of malignant tumors, the mutational burden of GBM remains low 10. Also, there are only very few high-frequency mutated driver genes like IDH1/IDH2 and promoter of TERT discovered in gliomas. The majority (i.e., 54-83%) of GBMs contain mutations in TERT promoter and are also commonly observed in oligodendrogliomas but rare in grade II or III astrocytomas. The IDH mutations are commonly observed in secondary GBMs (about 80%) and grade II-III diffuse gliomas (about 65-80%), while only about 5% of primary GBMs carry this type of mutation 11. IDH1 mutations are more commonly (>90%) observed in diffuse gliomas than IDH2 mutations, but are generally mutually exclusive 12.

The relationship between gene mutations and transcriptome remains the main focus of this study. Currently, there are no systemic analyses on the genotype-phenotype association. Thus, the potential relationship between the genotype and genetics, and clinical as well as pathologic features was elucidated via several statistical models in diffuse gliomas. Additionally, for clinical utility, we assessed the contribution of genomics, transcriptome and clinical variables for predicting survival. We also integrated all these variables for developing a prognostic model with high accuracy.

## Materials and Methods

### Data collection

The TCGA RNA-seq data, mutation data and phenotypic data of gliomas were collected with R (version 3.5.1, https://www.r-project.org) package *TCGAbiolinks*^13^. The raw count data of RNA-seq are normalized with R packages *DESeq2* and *preprocessCore*. The gene mutation data were collected, matched to patients and analyzed according to the description of the manual of *TCGAbiolinks*. The driver genes of diffuse gliomas were selected from *intOGen* (https://www.intogen.org). The gene mutation data were processed with R package *maftools*. The potentially harmful mutations including missense, nonsense, nonstop mutations, frameshift deletions and insertions were selected to perform further analysis. Only adult patients were selected for the analysis.

### Variable selection and classification

The multi-omics TCGA glioma dataset contains multi-dimensional information of each patient. The following are the important parts selected for the study. The selected variables were classified into five categories: (1) gene mutations: driver genes that are mutated in at least 5 patients, (2) gene expression profiles: transcriptomic data obtained from high-throughput sequencing of gliomas, (3) demographic variables including gender and age, (4) cytogenetics: 06-methylguanine-DNA methyltransferase (MGMT) promoter methylation status, Chr7gain and Chr10loss, 1p19q co-deletion status, TERT promoter mutation status, and (5) pathology: Verhaak's transcriptome subtypes, ABSOLUTE purity, histology, and WHO grade. According to the classifications of TCGA, LGG is defined as WHO grade II and III gliomas with several histological types including astrocytoma, oligodendroglioma, and oligoastrocytoma.

### Statistical model and analytical method

All statistical analyses were performed using R. Models and the algorithms in this study were derived from Gerstung M. et al^14^. The overall survival of patients was defined as the interval after glioma diagnosis till the last follow-up period or death. Cox regression model was built based on the overall survival of the patients, and the consistency between observed and predicted risk was evaluated using Harrell's C-statistic ranging from 0.5 to 1. The higher the Harrell's C-index value is, the better the predictive power of the specific prognostic model is. In survival analysis, the accuracy of a given variable in predicting the survival was tested using Cox regression with 5-fold cross-validation. The survival for patients was assessed with Kaplan-Meier estimates and differences in survival were compared by the log-rank tests. All statistical tests were two-sided, and a P-value of <0.05 was considered to be statistically significant.

## Results

The information regarding the two cohorts of 126 GBM patients and 481 LGG patients from the TCGA glioma dataset was collected. The selection criteria were described in the Methods section and data compositions were shown in **Figure S1**. The clinical characteristics of the two cohorts were shown in **Table S1**.

### Global gene expression is correlated with driver gene mutations, cytogenetic alterations as well as clinicopathologic features

**Figure 1:**
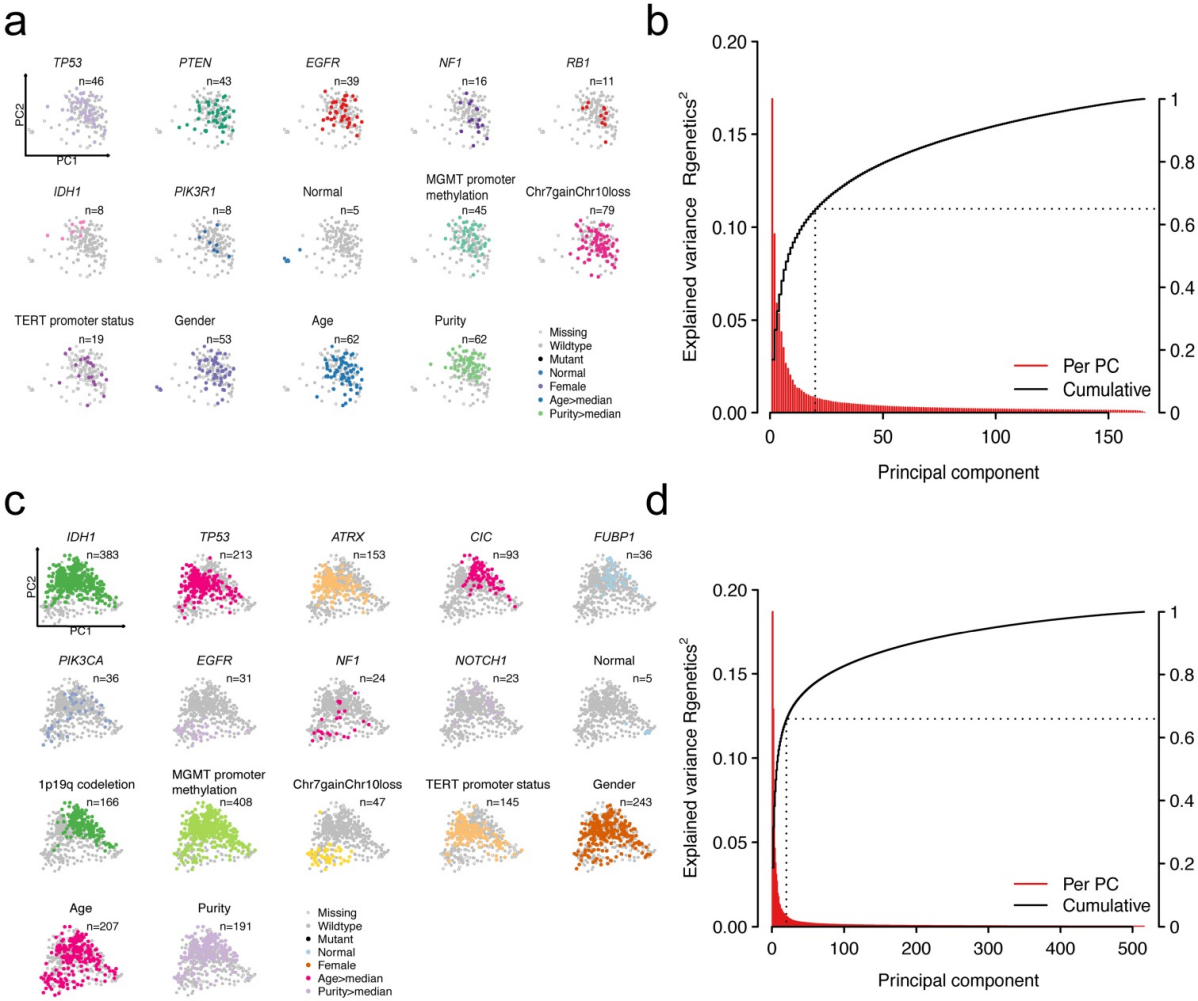
Patterns of genetic alterations and transcriptome in GBMs or LGGs. Scatter plots showing recurrent mutations, cytogenetic alterations as well as clinicopathological features that overlay the first two principal components (PCs) in GBMs (a) or LGGs (c). Each dot in the scatter plots resembles a patient. Explained variance of transcriptome by PCs in GBMs (b) or LGGs (d).

Heterogeneity in patients could be partially explained by heterogeneity in gene expression. A number of factors could explain the variations in gene expression. We mainly focused on the impact of mutations on driver genes to the expression profiling. Other factors including age, gender, and clinicopathologic features were also considered. To extract the main features of transcriptomic data, we implemented principal component analysis (PCA)-based dimensionality reduction analysis. The first two principal components (PCs) were displayed to explain the largest variance for each patient. We mapped different features on them by using different colors (**Figure 1a** and** Figure 1c**). For GBMs, the first two PCs could explain 16.8% and 9.6% of the total variability in gene expression (**Figure 1b**). For LGGs, the first two PCs accounted for 18.7% and 12.9% of the total variance (**Figure 1d**). Cumulatively, the first 20 PCs explained 65% and 66% of the total variance in GBMs and LGGs, respectively.

Visualization of patients by dimensionality reduction (PC1 and PC2) showed intriguing results genetically and cytogenetically. Alterations and other clinicopathologic features have distinct clustering directionality in the vector space of the first two PCs, suggesting that these alterations are associated with global gene expression changes. More specifically, the GBM patients with NF1 and IDH1 mutations were in opposite directions, suggesting the differences in the transcriptomic patterns of these two subtypes of patients (**Figure S2a**). For LGG patients, patients with IDH1 mutations or 1p/19q codeletion have similar PCA projections, indicating the similarity of the transcriptomic pattern (**Figure S2b**). As expected, the observed PCs reflected a continuum of changes in the expression and also clearly separated normal samples from tumor samples (**Figure 1a** and** Figure 1c**). These results showed that heterogeneity in genetic compositions partially explains the transcriptomic heterogeneity.

### Deconvolution of the interactions between driver mutations and gene expression via a linear model

We found that genetic alterations showed association with distinct changes in the transcriptomic profiles or specific gene expression. However, these relationships were not clearly understood yet. Thus, we deconvoluted these associations via a mathematical model. A multivariate linear model was used to measure the association of gene expression profiling and multiple predictors including the driver gene mutations, cytogenetic alterations, *etc.* Briefly, the multivariate linear model enables us to identify the gene expression changes induced by a specific alteration while controlling other confounding variables.

**Figure 2:**
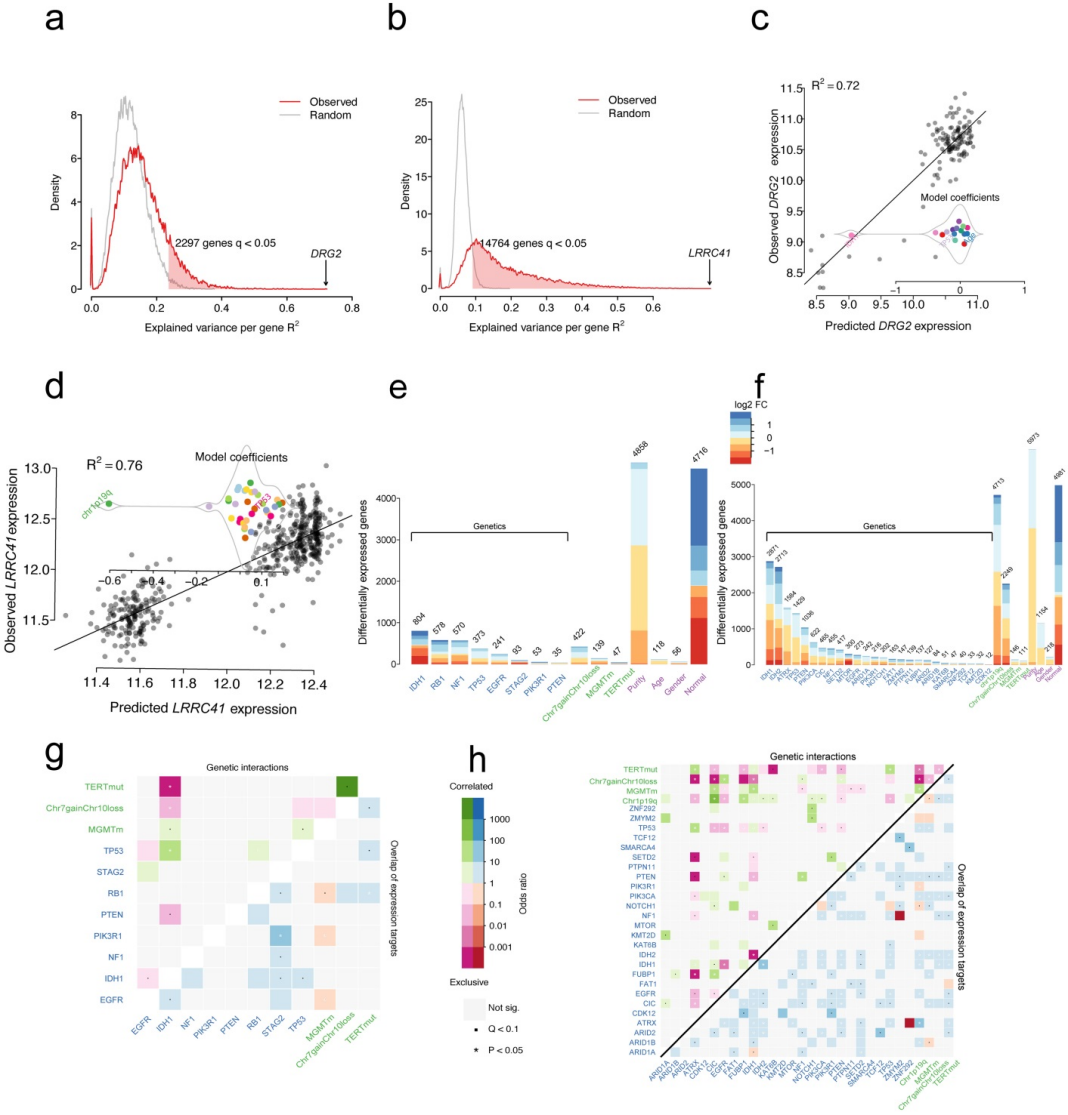
Distribution of variance explained by mutations and cytogenetic alterations in GBMs (a) or LGGs (b); Red line indicates genes with altered expression in association with genetic alterations and the grey line indicates genes with altered expression associated with all variables. Scatter plot of expression predictions for *DRG2* gene or *LRCC41* gene versus the observed expression values in GBMs (c) or LGGs (d); Model coefficients showed changes in the expression levels in association with specific alterations. The number of genes that statistically changed the expression associated with variables in GBMs (e) or LGGs (f) (moderated F-test; FDR < 0.05), MGMTm stands for *MGMT* promoter methylation, TERTmut stands for *TERT* promoter mutation; Logarithmic expression fold change (FC) is indicated by color. Heatmap displaying pairwise mutations (odds ratio; upper triangle) and overlapped target genes associated with each mutation (lower triangle) in GBMs (g) or LGGs (h).

The transcriptome of glioma is globally perturbed by genetic and cytogenetic driver mutations. After correction for multiple hypothesis testing (FDR-adjusted moderated F-statistic < 0.05), the expression levels showed that 2297/18429 (12%) and 14764/18429 (80%) genes were significantly associated with at least one driver mutation in GBM (**Figure 2a**) and LGG (**Figure 2b**), respectively. Genomic alterations accounted for at least R^2^=23.6% of the observed inter-patient gene expression variability in GBMs, and this explains at least the R^2^=9% of the total variance in LGGs (**Figure 2a** and** Figure 2b**). For GBMs, the strongest association reached R^2^=72% between mutations and expression changes for the gene *DRG2* (**Figure 2c**). The observed variability can be largely explained by the presence of IDH1 mutations, leading to the downregulation of *DRG2* gene expression. For LGGs, the strongest association reached R^2^=76% between mutations and expression changes for the gene *LRRC41* (**Figure 2d**). The presence of 1p19q co-deletion might explain the downregulation of *LRRC41* gene expression.

The multivariate linear model identifies the set of gene expression changes associated with a specific driver gene mutation from a mutation-centric perspective. As shown in **Figure 2e** and **Figure 2f**, each gene mutation showed an association with a specific set of targeted gene expression changes. For example, *IDH1* mutations showed an independent correlation with the expression levels of 804 genes, whereas *PTEN* mutations showed association only with altered expression levels of 35 genes in GBM samples. Similarly, in LGG samples, *IDH1* and *IDH2* mutations were independently correlated with most of the gene expression level changes (2871 genes and 2713 genes, respectively). Compared with normal samples, 4716 and 4981 genes changed the expression in GBM and LGG samples without being attributable to a distinct driver mutation (**Figure 2e** and** Figure 2f**). Furthermore, the differentially expressed genes (DEGs) that are induced by gene mutations, cytogenetic alterations or other features were mapped into chromosome locations. For driver gene mutations or cytogenetic gene mutations, the number of DEGs per chromosome broadly followed the gene density on the autosomes (**Figure S3**). However, the DEGs induced by *EGFR* mutations were located on 19 chromosomes (75/241, 31.1%), while those induced by *TERT* promoter mutations were located on 6 chromosomes (19/47, 40.4%) in GBM samples (**Figure S3a**). A similar pattern in LGG samples was not found for these mutations. For chromosome (Chr) 7 gain and chromosome 10 loss alteration, the largest share of expression changes occurred at the deleted or amplified genomic locus, resulting in the altered gene dosage in patients with LGGs and GBMs. For 1p/19q co-deletion, the DEGs tend to locate to 1 and 19 chromosomes in LGGs (**Figure S3b**). The sex-specific effects are predominantly localized to the X and Y chromosomes. These results suggested no tendency of chromosome localization of IDH mutations in both LGGs and GBMs.

For the driver gene itself, the *NF1, RB1, PIK3R1,* and* TP53* mutants in patients demonstrated significantly lower levels of expression when compared with wild-type in GBM and lower expression levels of *FUBP1, NF1, PTEN,* and *IDH1* in mutant patients in LGG (**Figure S4**). In contrast, the expression level of *EGFR* was highly expressed when mutated (**Figure S4**). The expression levels of driver genes also provided evidence on the pathogenesis of gliomas.

### Mutation profiling and transcriptome predict the clinicopathologic features of patients

**Figure 3:**
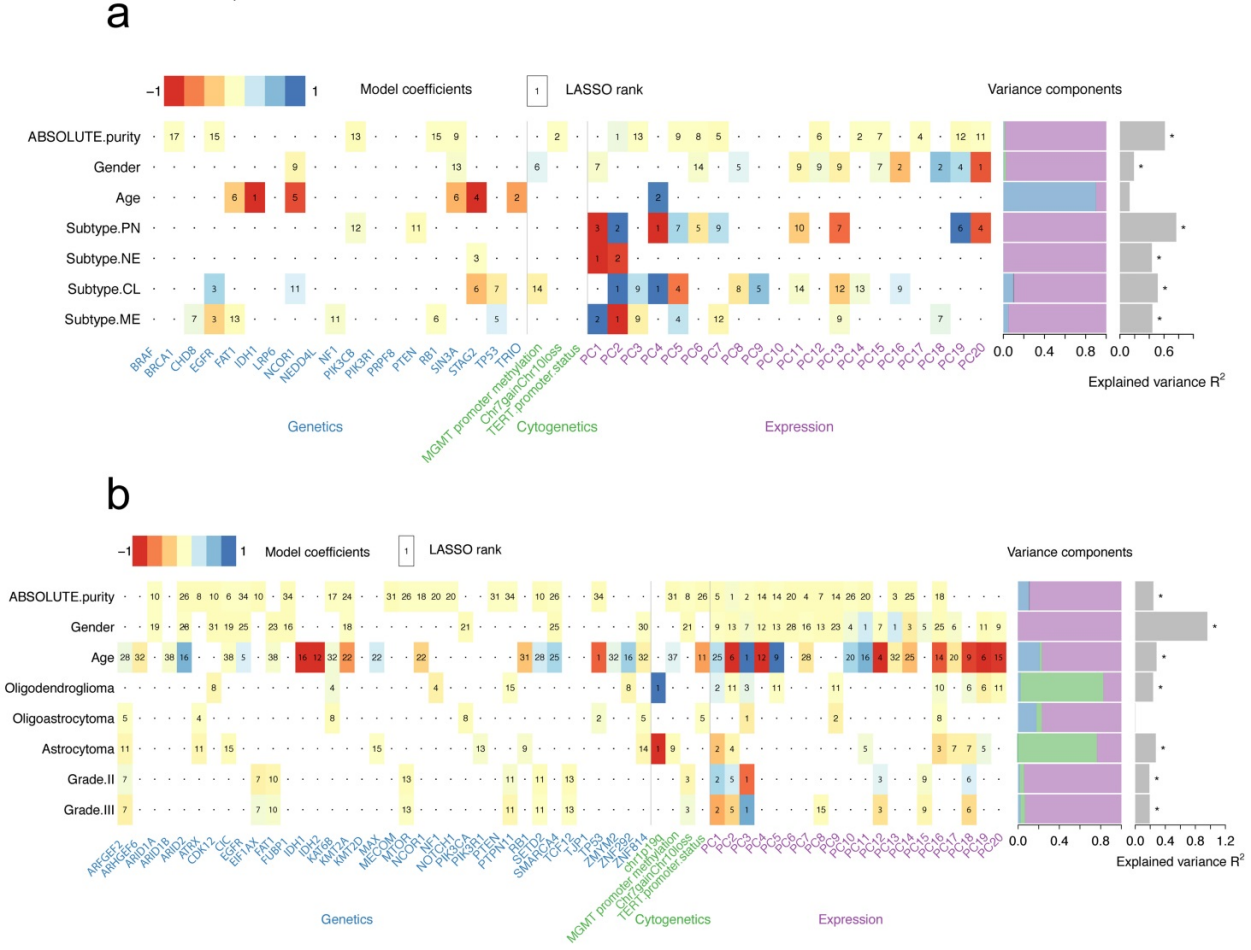
Heatmap summarizing the model coefficients for clinicopathological features in GBMs (a) or LGGs (b). LASSO-selected (least absolute shrinkage and selection operator) coefficients are colored and ranked by numbers. Numbers in bold fonts indicate highly significant coefficients. Variance is explained by genetic, cytogenetic and expression variables as shown in the bar plot. The R^2^ of models that are larger than zero indicating a margin of more than one s.d. is denoted with a star.

We have explored the association between genetic alterations and expression profiling and analyzed the possible underlying mechanisms. Next, a model was set to demonstrate the association between all genetic/expression factors with clinicopathological features. To explore these associations, a generalized linear model was used to quantify the association of common genetic and cytogenetic alterations, as well as the first 20 PCs of the transcriptome with several clinicopathological features (**Figure 3**). For example, a strong predictive value for purity in GBMs was found and the two strongest predictors included PC1 from gene expression data and presence of Chr7 gain and Chr10 loss (**Figure 3a** and **Figure S5**). The optimal model demonstrated a predictive accuracy of R^2^ = 61.2%. The transcriptome subtypes of GBMs reflected the transcriptomic pattern of GBMs. PC1 and PC2 were considered as the two strongest predictors for mesenchymal (MES) or neural (NE) subtype, while PC2 and PC4 for classic (CL) or proneural (PN) subtype (**Figure 3a**). Predicting the PN subtype demonstrated the highest predictive accuracy with R^2^=76.6% (**Figure S5**). However, no strong predictive effects of these variables to features including age, grade as well as histology in LGGs were observed (**Figure 3b** and **Figure S6**). Consistently, we found the association between the presence of 1p19q codeletion and oligodendroglioma (**Figure 3b**). We also found the fundamental differences in predictors for different histology subtypes, suggesting various molecular mechanisms.

### Predictive power of expression, mutations and clinical data in prognosis

**Figure 4:**
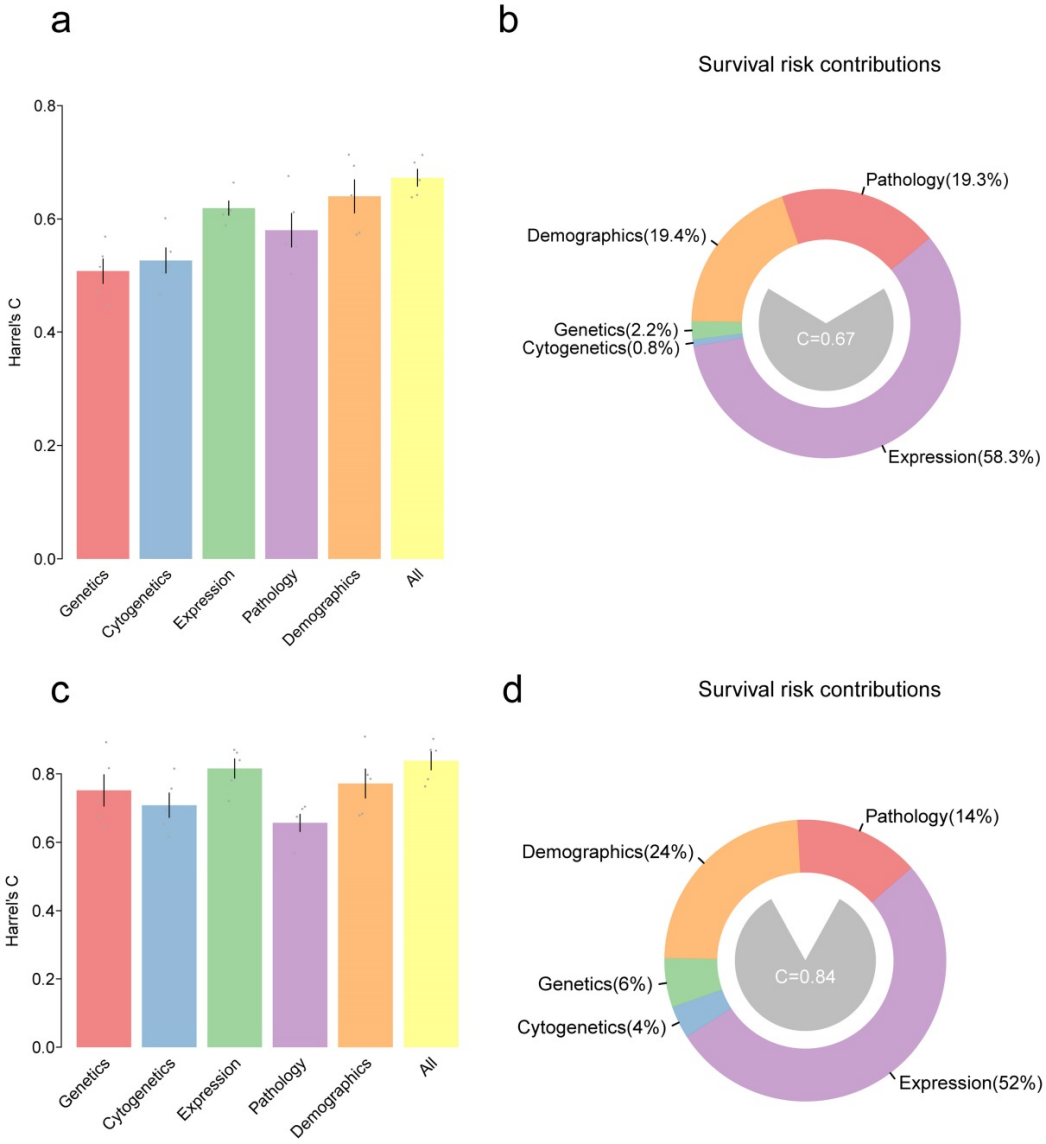
Bar plot showing Harrel's C-statistic for multivariate Cox proportional hazards models to predict the overall survival in GBMs (a) or LGGs (c); Grey points denote the C-index of 5-fold cross-validation prognostic models and error bars represent the mean s.d. Distribution of variables that contributed to the survival risk in GBMs (b) and LGGs (d).

We have developed models for predicting the clinicopathological features. Furthermore, as predicting survival outcomes in patients is a key issue for clinicians, models were finally set out to predict the prognosis. Here, a multivariate Cox proportional hazards model was used to predict overall survival (OS) in GBM and LGG patients using variables in all the five types of features (genetic, cytogenetic, transcriptomic, demographic and pathologic features). Harrel's C-statistic was used to evaluate the predictive power of the model. Moreover, all variables in a given type were integrated into one and hoped to improve the predictive power of the model. As shown in **figure 4**a, for GBM patients, accuracy of the model by using genetics alone was C=50.8% and accuracy of the model by using cytogenetics alone was C=52.6%, and these were inferior to that obtained by using the expression data (based on the first 20 PCs) with C=61.9%. The combination of variable classes resulted in the highest prediction of the accuracy of C=66.3%. For LGG patients, the accuracy of expression, demographics, and pathology alone was C=81.5%, 77.1%, and 67.1%, respectively. The combination of all variables slightly improved the predictive accuracy of the model to C=84.5% (**Figure 4c**). Notably, the gene expression contributed mostly to the risk estimation in both GBM (58%) and LGG (52%) (**Figure 4b** and **Figure 4d**).

Next, the potential key factors that contributed to most of the prognosis were identified. Therefore, random forests (RF)-based survival analysis was performed (**Figure S7**). According to the random forest model, the variables with high importance are considered as major contributors to the outcomes. Age was considered as the major factor for the survival risks in GBMs or LGGs. For GBMs, PC17 and PC2 were the major prognostic factors in the expression class, and purity was regarded as a major prognostic factor in the pathology class, while IDH1 mutations were considered as significant prognostic factors in the genetics class (**Figure S7a**). Similarly, in LGGs, PC2, PC3, PC1, and PC18 in the expression class contributed to most of the prognosis. IDH1, and EGFR mutations in the genetics class and the presence of chr7 gain and chr10 loss in the cytogenetics class were also the major contributors of survival risk (**Figure S7b**).

## Discussion

Like other tumors, the clinical features and pathological features were used for predicting the prognosis of gliomas for many years. Increased data regarding transcriptome and genome provides new insights on the disease and also helps us to establish a prognostic prediction model with high accuracy. Gliomas are a group of diseases with high heterogeneity. The TCGA consortium has performed high-dimensional molecular profilings of nearly 600 gliomas and established glioma molecular subtypes 15, 16. These molecular subtypes provide evidence for understanding tumor biology and are also associated with significant patient outcomes. Previous studies have identified the associations between tumor subtypes, in which the IDH-mutant gliomas were associated with cytosine-phosphate-guanine (CpG) island methylator phenotype (G-CIMP) 17. However, the relationships between the expression profiles and mutations were not well understood. Therefore, in this study, an *in silico* method was employed to deconvolute the relationships between genetic alterations, transcriptome, clinicopathological features and patient outcomes using the TCGA data on gliomas. Our study confirmed some known mechanisms and also revealed some striking observations.

Previous studies have revealed that gene expression is affected by many confounding factors. A linear model was used to measure the correlations between the expression and the mutations by controlling other confounding variables. These findings revealed that the global transcriptome of gliomas is perturbed by driver mutations and cytogenetic alterations. Also, several transcripts that altered the expression associated with at least one mutation were identified. Compared with GBMs, the transcriptome of LGGs were profoundly affected by more genes that changed the expression. Moreover, the affected genes varied in numbers across different driver mutations. Indeed, for both GBMs and LGGs, the IDH mutations induced most of the gene expression changes, confirming the core role of IDH mutations in glioma pathogenesis 18, 19. Moreover, our data revealed a difference in the impact of driver mutations on GBMs or LGGs, which included RB1 mutations for GBMs and PTEN, ATRX, and CIC mutations for LGGs. Additionally, other factors including tumor purity and age could also affect the gene expression levels. However, limited overlapping was found in genes that altered the expression induced by different mutations or other factors, suggesting that these variables acted in a functionally distinct manner. These results indicated the complexity and heterogeneity of gliomas.

Although our methods of model-building did not provide us the exact mechanism on the level of molecular biology as to how the mutation of a certain driver gene affects the expression of other genes, our work showed a connection between mutation and transcriptome, providing a theoretical basis for further experiments in gliomas. By taking DRG2 as an example, significantly lower expression in GBM patients with IDH1 or TP53 mutation, and significantly lower expression level of LRRC41 were observed when there is 1p/19q co-deletion in LGG patients. The differences here indicate fundamental heterogeneity regarding the molecular mechanism on how the driver gene mutations influence transcriptome between GBMs and LGGs. Most of the driver genes of glioma showed decreased expression when mutated but not for EGFR. As a known oncogene of glioma, EGFR overexpression usually occurs as a result of copy number amplification, while in other cases where there is no copy number variation, EGFR has the ability for ligand- independent activation by some point mutations or frame-shifting insertions/deletions. Thus, the activation of EGFR in glioma can be achieved by multiple independent pathways, whether co-exists or not 20, 21.

Chromosomal distribution of affected genes by genetic factors seems to be extensive and random, showing no concentration on any chromosomes, except in a few cases. Structural variation of chromosomes like chr7 gain and chr10 loss affects the genes on these, which remained obvious, and it is the same for co-deletion of 1p/19q. Point mutations of *EGFR*, promoter of *TERT*, *PI3KR1*, and *KMT2D* affects genes on chromosomes 19 and 6, which are not the chromosomes they originated from. Coincidently, chromosome 19 includes a lot of genes that are directly associated with the malignancy of glioma^22^, and chromosome 6 includes many known tumor suppressors like TERT suppressor^23^. Somehow these mutations have a profound impact on the transcription of genes that are even far away from the physical location, and our work provides a lead to further analyze the exact mechanism.

All the variables described in this study demonstrated significant effects on the transcription profile and are probably considered to be the most essential factor for the prognosis of glioma patients. It is not difficult to understand the importance of certain gene expression changes in the evolution of gliomas, but surprisingly, our results showed that transcriptome contributes the most in predicting the survival of patients when compared with other types of factors in the model, no matter whether in LGG or GBM. The transcription profiles contributed to 58% in the GBM model, and 52% in the LGG model. The model achieved the highest accuracy when all the variables were combined, indicating that other variable types like cytogenetics, demographics, and pathology still contained some information related to the survival of patients. In the RF-based analysis of patient survival, age is regarded as the most important variable of all in both LGGs and GBMs. For all pathological variables, histology demonstrated the highest importance in LGGs, and purity index for GBMs. Our results indicated that glioma purity has great value in predicting the prognosis, which was also pointed out by other researchers 24-26 but is not widely used.

However, there are some limitations to this study. Firstly, analysis within the TCGA cohort was performed, in which both the training set and validation were included. N-fold cross-validation and bootstrap methods were used to reduce bias from a single dataset to as minimally as possible. It would be better if we had another external dataset to further confirm some of the results. Secondly, potential confounding variables that alter the prognosis of patients existed, which included the race of the patients, treatment, and gene methylation status. As a retrospective study, it is not possible to fully eliminate the bias during the patient selection process.

## Conclusion

In conclusion, the genetic and phenotypic relationship in glioma patients was generated, and a model based on the combination of cytogenetics, transcriptome, histology and demographic data was made to accurately predict patients' prognosis in both GBMs and LGGs. The transcriptomic data is considered to be the most important of all, but the model needs all variables to achieve the highest efficacy. All data should be taken into consideration to ensure accurate prediction in glioma patients in the future.

## Supplementary Material

Supplementary figures and table.

## Abbreviations

CL: classic
GBM: glioblastoma multiform
G-CIMP: cytosine-phosphate-guanine island methylator phenotype
LASSO: least absolute shrinkage and selection operator
LGG: low-grade glioma
MES: mesenchymal
MGMT: 06-methylguanine-DNA methyltransferase
NE: neural
PCA: principal component analysis
PN: proneural
TCGA: The Cancer Genome Atlas
