# Supplementary material for: Integrating Genomic Data with Transcriptomic Data for Improved Survival Prediction for Adult Diffuse Glioma
Source: J Cancer. 2020 Apr 6;11(13):3794–802. doi: 10.7150/jca.44032 (PMC7171505; doi:10.7150/jca.44032)
Supplement: Supplementary file 1 — Supplementary figures and table. [file jcav11p3794s1.pdf]

Supplementary materials

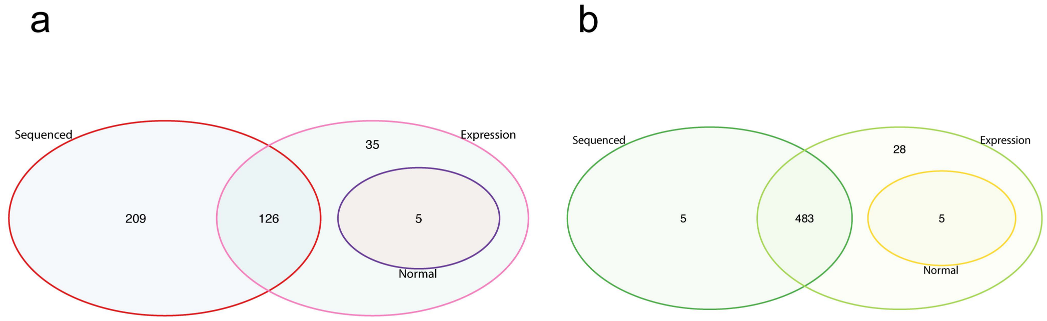

Figure S1

Venn diagram showed data compositions of samples in our study (GBMs (a) and LGGs (b)). Samples with sequenced and transcriptome data were selected. 2 (age was 14 and 17 years) out of 483 patients were excluded in LGG cohort for further analysis.

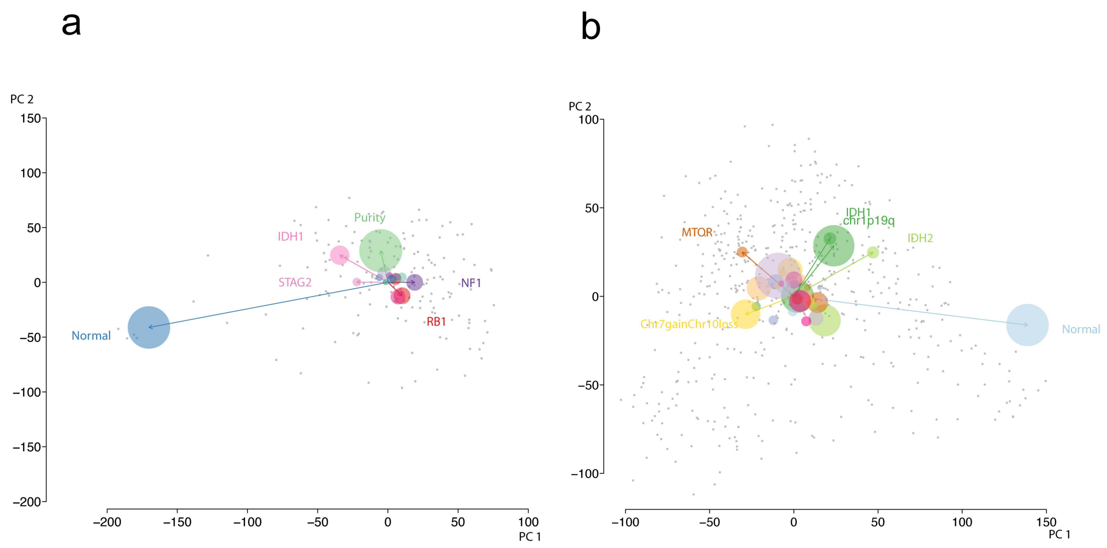

Figure S2

PCA projections of variables in the first two PCs in GBMs (a) or LGGs (b). The size of points denoted the number of targeted genes that changed in expression associated with these variables.

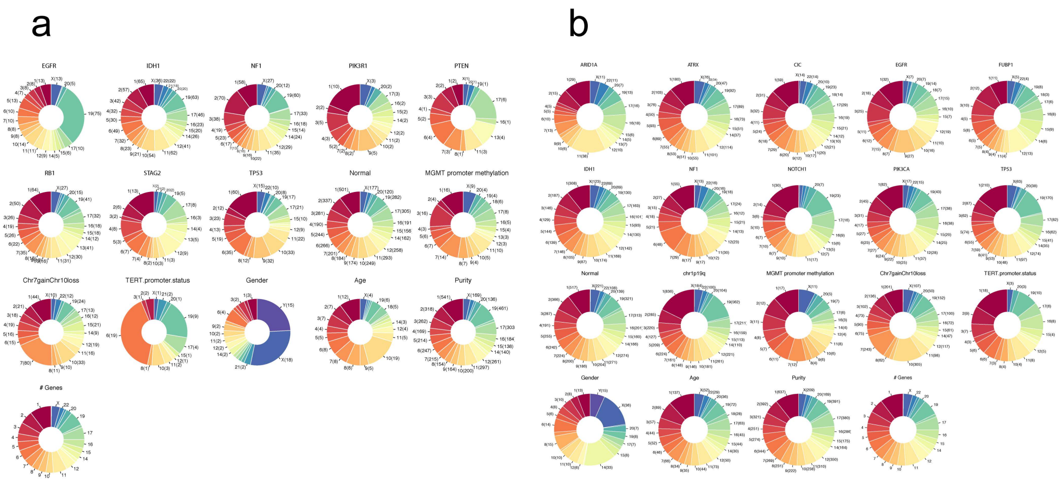

Figure S3

The distribution of genes that changed in expression significantly in the chromosome location in GBMs (a) or LGGs (b).

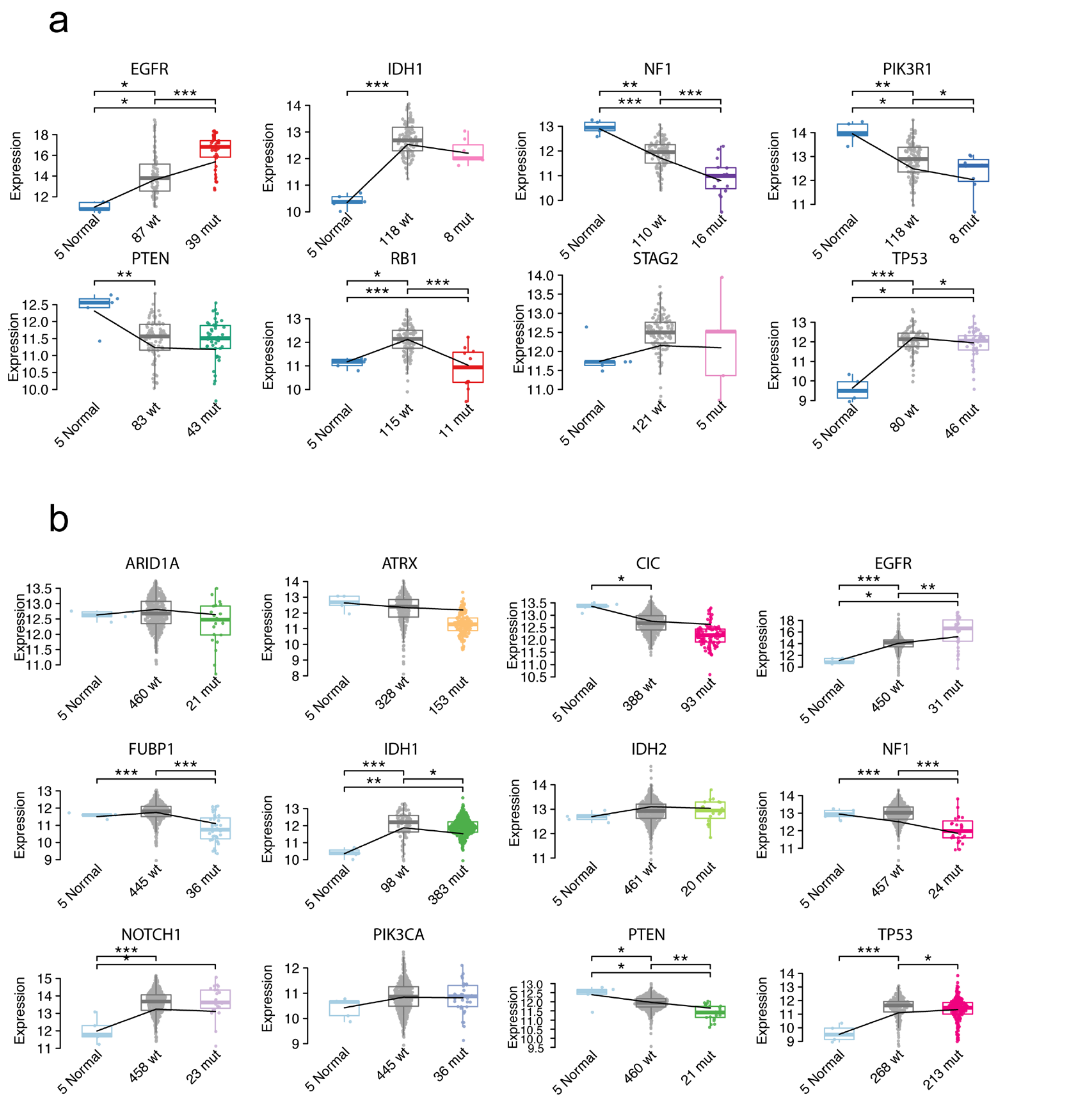

Figure S4

Expression levels of driver genes in GBMs (a) or LGGs (b). The lines represented the expression predicted by the linear model. Stars indicated significance, adjusted to the number of shown comparisons. (***P<0.001, **P<0.01, *P<0.1; FDR-adjusted moderated t-test)

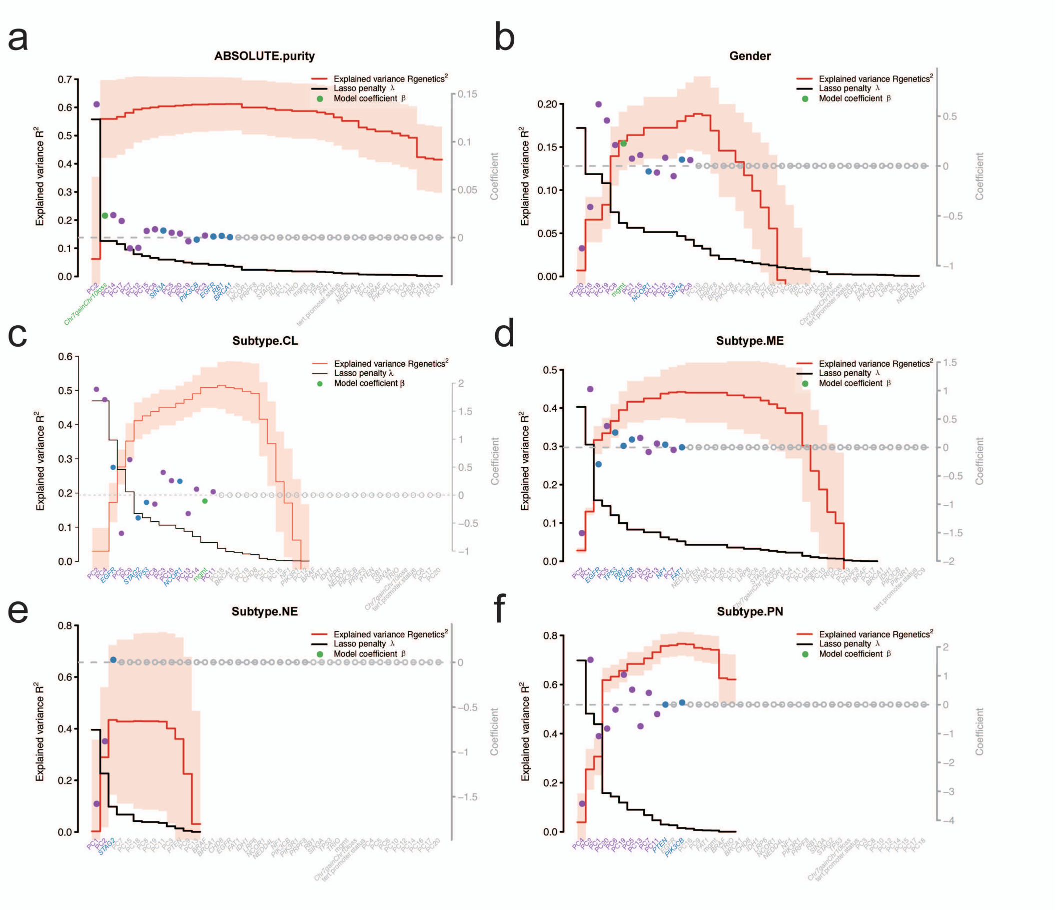

Figure S5

Variance explained by selected variables ordered by their model coefficients in a LASSO penalized model in GBMs. The optimal model used the largest explained variance R2.

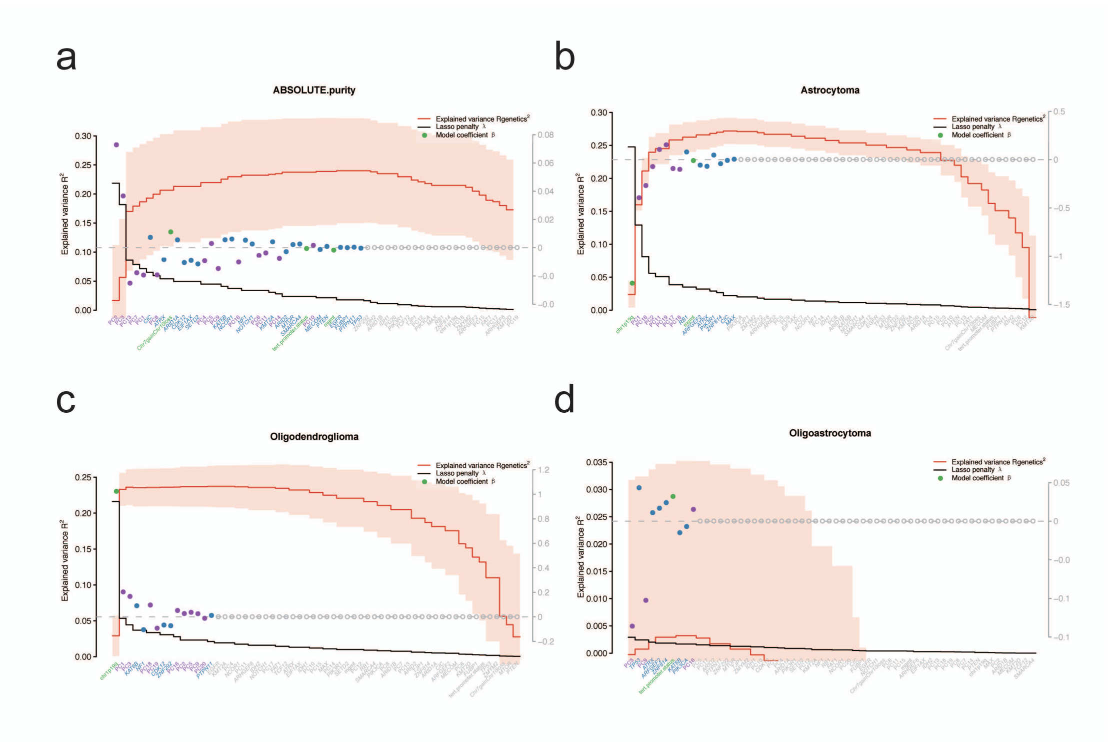

Figure S6

Variance explained by selected variables ordered by their model coefficients in a LASSO penalized model in LGGs. The optimal model used the largest explained variance R2.

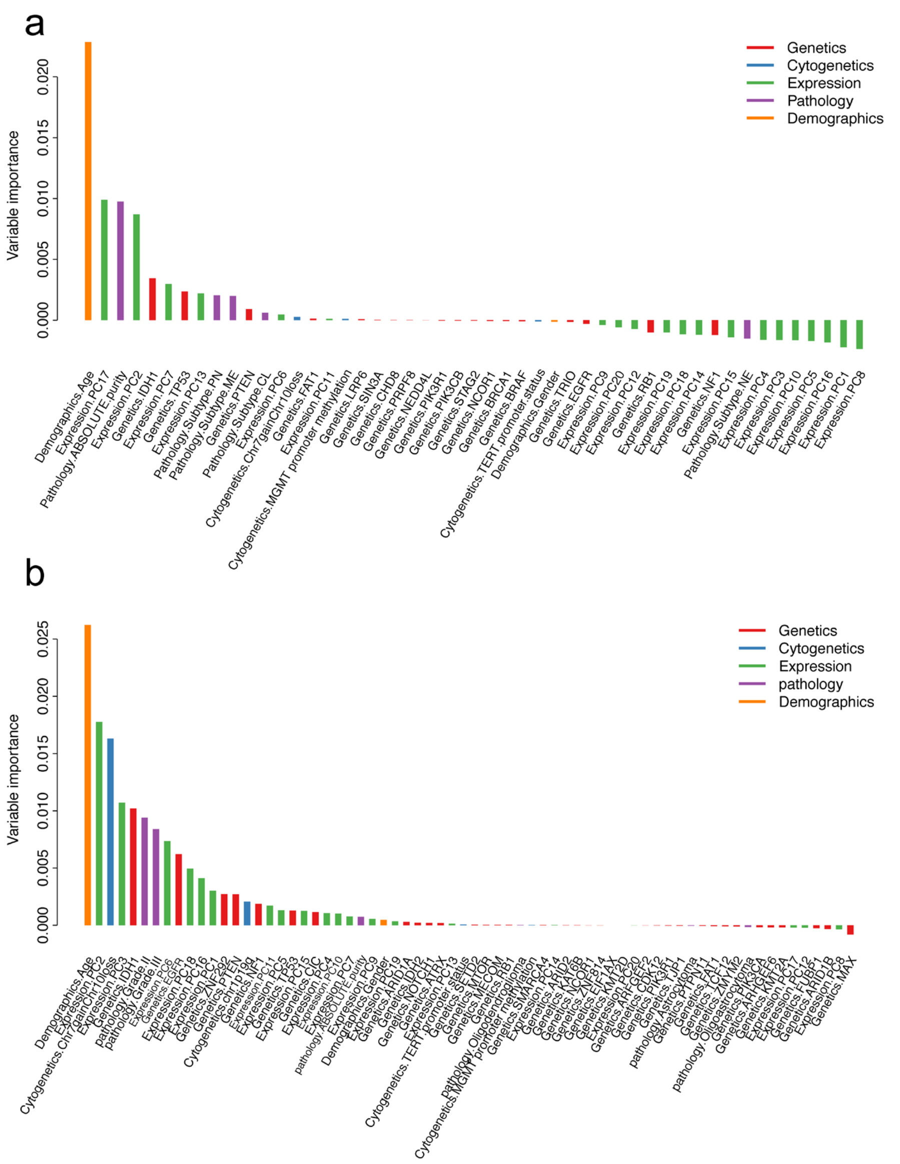

Figure S7

Bar plot showed the variable importance of random forest (RF)-based survival analysis in GBMs (a) or LGGs (b).

Characteristics	Glioblastoma	Low-grade glioma
**No. of patients**	126	481
**Age at first diagnosis**	60 years median; 21-89 years range; 1 missing	41 years median; 19-75 years range; 56 missing
**Gender**		
Female	50	240
Male	75	185
NA	1	56
**Transcriptome Subtypes**		
Proneural	16	213
Neural	6	93
Classic	37	29
Mesenchymal	55	28
NA	12	118
**ABSOLUTE purity**	0.79 (0.17-1)	0.76 (0.16-1)
**1p/19q codeletion**	0	166
**MGMT promoter methylation**	45	408
**IDH1 mutations**	8	383
**TERT promoter mutations**	19	145
**Chr7gain and 10 loss**	79	47
**WHO Grade**		
II	-	198
III	-	227
IV	126	-
NA	0	56
**Histology**		
Oligodendroglioma	-	163
Oligoastrocytoma	-	106
Astrocytoma	-	156
GBM	126	-
NA	0	56

Table S1. Clinical characteristics of patient information in this study
